# The “Altitudinal Anton’s Syndrome”: Coexistence of Anosognosia, Blindsight and Left Inattention

**DOI:** 10.3233/BEN-2012-110224

**Published:** 2013

**Authors:** A. Carota, F. Bianchini, L. Pizzamiglio, P. Calabrese

**Affiliations:** ^1^Neurocenter GSMNGenolier ClinicGenolierSwitzerland; ^2^Department of PsychologyUniversity of Rome La SapienzaRomeItaly; ^3^Neuropsychological LaboratoryIRCSS Santa Lucia FoundationRomeItaly; ^4^Faculty of Cognitive PsychologyUniversity of BaselSwitzerland

**Keywords:** Cortical blindness, altitudinal hemianopia, anosognosia, attention blindsight, right hemisphere damage

## Abstract

We describe a 69-year-old patient with superior altitudinal hemianopia who contentiously denied having any visual impairment after stroke in the lower banks of both calcarine fissures. Although the patient did not produce intentional responses to visual stimuli in the blind fields, he showed reduced reaction times to stimuli presented in the inferior visual fields when they were primed by identical stimuli in the superior blind fields. Furthermore he showed left extinction to the double stimulation and delayed reaction times for left unprimed stimuli in the inferior fields. Based on these findings we discuss the possibility that blindsight and right hemisphere damage might be both necessary conditions for denying bilateral blindness.

## 1. Introduction

Seeing and being aware of seeing require intact visual perception and adequate insight of perception itself.

A defective match between visual perception and visual awareness occurs in patients who have extensive bilateral lesions of the geniculo-calcarine pathways and contentiously deny blindness. This disorder is called "Cortical Blindness", "Anton-Babinski syndrome", or "Anton's syndrome" [1].

We were interested in assessing whether, in patients with bilateral loss of visual fields (such as occurring in Anton's syndrome), denial of blindness might be somehow related to the presence of unconscious perceptual processes (e.g. blindsight), which have been largely documented to occur in patients suffering from unilateral occipital lesion and contralateral loss of visual field (hemianopia) [2,3]. We were also interested in determining whether, analogously with the anosognosia of hemiplegia and hemianopia, patients who deny bilateral blindness also present associated signs of right hemisphere dysfunction.

To our knowledge, there are no previous studies showing left spatial neglect in patients with Anton's syndrome, mainly because the complete blindness prevents the use of explicit paradigms, at least in the visual modality. In the literature there are only few detailed case studies of patients with altitudinal hemianopia and bilateral calcarine stroke [4,5]. However, in these reports, patients did not show anosognosia for the visual impairment and confabulations, nor signs of spatial neglect or visual agnosia.

Here we report the case of MP who presented the clinical features of Anton's syndrome (bilateral occipi-

ISSN 0953-4180/13/$27.50 © 2013 – IOS Press and the authors. All rights reserved tal damage, blindness and denial of visual loss with confabulations), which were limited to the bilateral upper visual field. We named this condition Altitudinal Anton's syndrome in analogy with the Anton's syndrome where confabulations and lack of awareness concern a deficit in the whole visual field.

This rare situation lead us to investigate whether anosognosia of a bilateral visual deficit coexists with left spatial inattention (assessed in the spared lower visual fields) and signs of unconscious perception (blindsight). Indeed, with an altitudinal visual loss, the presence of attention blindsight could be demonstrated by an experimental paradigm that measures the effects of primes in the blind (upper) visual fields on the responses to probes in the intact (lower) visual fields [6–8].

## 2. Patient history

A 69-year-old right handed Frenchman (MP), who had 12 years of education, was examined 6 months after a bilateral occipital stroke. MP was known for arterial hypertension and hypercholesterolemia. Left vertebral artery occlusion and diffuse vertebral atheromatosis were retained as the cause the stroke. MP was a retired worker who was completely autonomous and had no history of memory, language or behavioral disturbances prior to the event.

The neurological examination showed abnormal upright posture (30° head extension with conjugate downgaze deviation), a superior altitudinal visual deficit to confrontation, without motor or sensory deficits. Formal assessment of MP's visual fields with Goldmann perimetry showed a superior, congruent, and bilateral superior altitudinal visual loss with relative macular involvement (Fig. 1A). Visual acuity was normal.

As visualized by MRI, the ischemic lesions were located bilaterally in the lower banks of the calcarine fissure (lingual, parahippocampal and fusiform gyrus, comprising Brodmann areas 17,18,19,27,30) (Fig. 1), in the superficial territory of the posterior cerebral arteries.

## 3. General cognitive and behavioral assessment

MP firmly denied any kind of visual deficit and often confabulated about his vision. For example, he repeatedly said that his sole problem with the eyes, was a "red eye", and that his eye had become red since he underwent an ophthalmological treatment for conjunctivitis. Actually the patient did not have conjunctivitis or other ocular signs. When MP was explicitly challenged about his loss of vision in the superior fields, he attributed it to reduced lighting conditions or he said that the examiner was systematically lying about moving hands, fingers or other stimuli, and that he was perfectly able to see anything and to watch television. MP's obstinacy in denying blindness of a large part of the visual field went to the point to threaten the medical team when nurses and doctors made comments about his head and ocular vertical movements, likening him to near blind person who adapts his residual vision to the objects. However, MP's anosognosia extended to other forms of visual impairment. For example, although MP bought the newspaper every day and vehemently insisted on being able to read it normally, when the examiner asked him to read the headlines aloud the patient showed the classical features of pure alexia. In that situation, confronted with the deficit, the patient used to justify himself saying that his glasses were not well adapted.

His score on the Edinburgh Handedness Questionnaire corresponded to the 30th %le for right-handedness. MP's digit span was 6 forward and 4 backwards.

The general assessment revealed impaired executive function (FAB score: 12/18; TMT A: 120" $<$ 1%le, 2 errors; TMT-B: unable), object (responsive naming 10C/30; confrontation naming 60C/105, animal naming 15C/23), colors (5C/10), and proper name anomia (celebrity faces: 4C/30 and famous monuments 8C/30), while object visual recognition and body-part identification (matching name to figures) were spared (70C/72 and 22C/22 respectively). MP showed pure alexia (letter-by-letter reading) and verbal and visual memory impairment (CVLT, trials 1–5: 20, $<$ 1%le, short and long free and cued recall: $<$ 1%le; Rey-Osterrieth complex figure, 30 min delay: 0/36$-$ $<$ 1%le), Although the patient showed no signs of visual agnosia or of left spatial neglect in various tests (bell cancellation, line bisection, recognition of embedded figures, copy of the Rey-Osterrieth complex figure), he favored a right-left strategy and showed left visual extinction with double simultaneous stimulation (stimuli presented in the inferior visual fields).

MP's anosognosia also extended to other cognitive deficits (i.e. memory or naming), but he confabulated only on his visual abilities, a fact that prevented any form of visual rehabilitation.

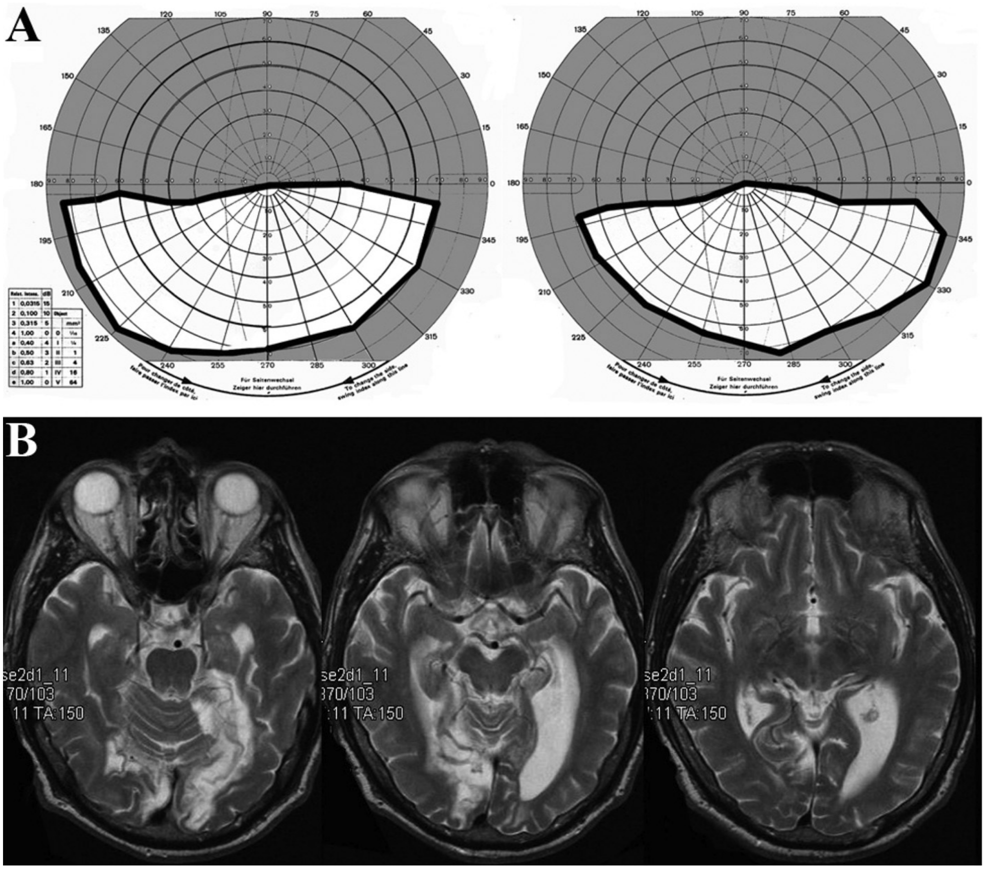

Fig. 1. Visual field assessment. A. The Goldmann perimetry showed superior, congruent, and bilateral superior altitudinal hemianopia with partial involvement of the macular vision; B. Axial T2-weighted images showed bilateral, almost symmetrical, chronic ischemic lesions, which are located in the occipito-temporal regions under the calcarine fissure, in the superficial territory of posterior cerebral arteries.

## 4. Experimental investigation

MP participated in two experiments, which took place in two different sessions. The tasks were administered by means of a Macintosh G5 powerbook computer (Apple) and constructed with PsyScope [9].

Both experiments consisted in responding as fast as possible by tapping a key at the appearance of a target stimulus. Reaction times (RT) were recorded for each trial. The stimuli, which consisted of colored diagonal crosses (width: 2.9°), were randomly projected onto a black background on a 21" color monitor refreshed at 85 Hz.

The patient viewed the screen from a distance of about 60 cm. His head was held in a constant position by means of a combined chin-forehead rest. The patient was instructed to fixate a white dot (1.9° diameter) in the centre of the screen during all the trials.

Stimuli were randomly presented in fixed positions of the superior (X:40° Y:40°; X:-40; Y = 40°) and inferior (x:40° y:-40; X:-40°, y:-40°) left and right visual fields. The inter-trial interval was randomly fixed at several values (2000, 2500, 3500, 4500 and 5000 msec). Each experiment was preceded by a 5-min training session.

In the *1st experiment* MP was exposed to 60 visual stimuli of 2 sec duration for each quadrant of the visual fields (total of 240 trials). Stimuli disappeared after key tapping. There was a 5-min pause after the first 120 trials.

In the *2nd experiment*, the same targets were randomly presented in two different conditions, that is, with and without priming. In the condition without priming, the stimuli were presented only in the inferior visual field locations (60 trials for each location for a total of 120 trials). In the condition with priming, the priming and the probe were identical stimuli (perceptual priming). In this condition, the trial consisted of a 1st priming stimulus of 20 msec duration, which was randomly presented in each of the two superior (blind)

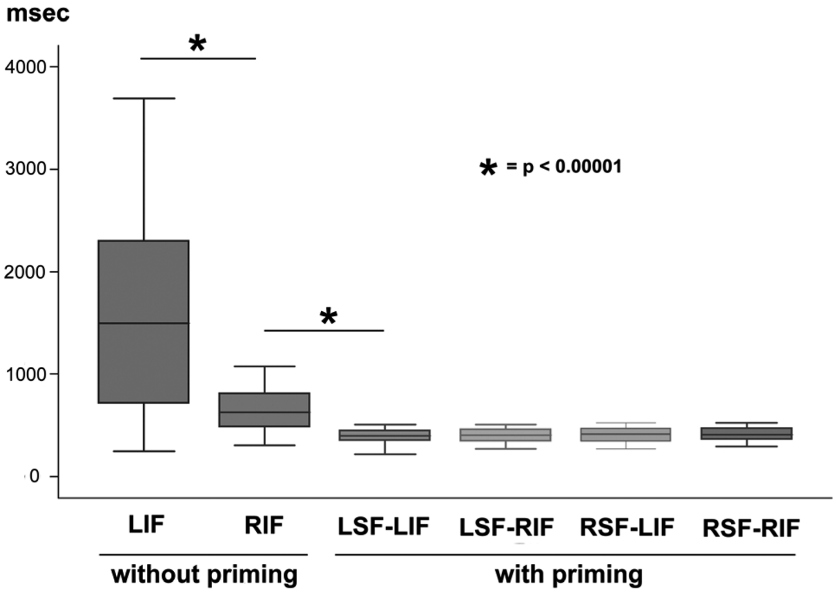

Fig. 2. Boxplots of RTs in conditions with and without priming. Boxplots of RTs in the second experiment. Note the significant differences between RTs in conditions with and without priming. *Condition without priming: LIF*: left inferior field; *RIF*: right inferior field; *Priming condition*: the first element indicates the location of the priming stimulus and the second element the location of the target stimulus. *LSF-LIF*: left superior field – low inferior field; *LSF-RIF*: left superior field – right inferior field; *RSF-LIF*: right superior field – left inferior field; *RSF-RIF*: right superior field-right inferior field.

visual hemifields and was followed at random intervals (200, 250, 300-msec) by a probe in each one of the inferior hemifields (60 trials with priming in the left superior field [LSF] and a probe in the left inferior field [LIF], 60 trials with priming in the LFS and a probe in the right inferior field [RIF], 60 trials with priming in the right superior field [RSF] and a probe in the LIF, 60 trials with priming in the RSF and a probe in the RIF).

There were a total of 360 trials in the second experiment (120 without priming and 240 with priming) with a 5-min pause after each 120 trials.

## 5. Results

In the *1st experiment*, the patient did not respond to any target (accuracy: 0%) in either superior field, whereas he responded to all targets (accuracy: 100%) in both inferior visual fields. At the end of the experiment, the patient reported that he had responded to all the visual stimuli he had seen and that he had never felt like he was missing a target. In the *2nd experiment*, in the condition without priming RTs (mean±1SD) were: LIF: 907 ± 860 msec, RIF: 630 ± 227 msec; in the condition with priming RTs (mean ± 1SD) were: LSF-LIF: 392 ± 60 msec; LSF-RIF: 383 ± 68 msec; RSF-LIF: 394 ± 67 msec; RSF-RIF: 417 ± 63 msec (Fig. 2).

The difference between RTs to stimuli presented in the LIF and RIF without priming, was significant (paired t-test: $p < 0.00001$), suggesting some form of left spatial inattention. The difference between the RT means in the condition without priming and in each condition with priming was also significant (paired t-test: $p < 0.00001$) suggesting the existence of a repetition perceptive priming effect caused by stimuli appearing in the blind fields on the probes of the inferior visual fields, irrespectively of the stimulus location, of either the primes or the probe. This last finding (unconscious interference by a stimulus appearing in the blind field on the reactions to stimuli processed in the spared visual fields) is generally considered an "attention-blindsight" phenomenon [8].

## 6. Discussion

We reported the first case of denial of a congruent superior altitudinal hemianopia (with relative macular involvement), a condition that we defined as Altitudinal Anton Syndrome. This syndrome corresponded to the bilateral selective involvement of the inferior lip of

Table 1
Visual cortical syndromes with and without anosognosia

| | Normal vision | Visual field loss | |
|---|---|---|---|
| *Spatial neglect-* | Awareness of seeing (*Normal vision*) | Awareness of not seeing (*Experience of blindness*) | Blindisght+: *feeling of continuity of the visual stream*<br>Blindisght-: *feeling of fragmentation of the visual stream* |
| *Spatial neglect+* | Unawareness of seeing (*Anosognosia of spatial neglect*) | Unawareness of not seeing (*Anosognosia of visual field loss or cortical blindness*) | Blindisight+/−: *Confabulations +/−* |

This table summarizes the syndromes that might occur as a consequence of trading off among blindsight, spatial neglect and anosognosia and a visual field deficit.

the calcarine area (including the occipital pole) with sparing of the superior lip.

We draw an analogy to Anton's syndrome as MP had bilateral calcarine lesions and showed impaired awareness of a visual deficit that was bilateral. Furthermore the visual disturbance was the only perceptive impairment and, although MP's anosognosia extended to other non perceptive deficits, his tendency to confabulate and to dismiss the deficit was remarkable only in the visual modality. Thus, for MP, denying blindness seemed to correspond to a specific form of anosognosia. MP's obstinacy in affirming that his vision was normal in spite of clear evidence for the contrary clearly reminded that form of anosognosia which is classically reported in individuals with Anton's syndrome [10]. Our results showed that MP's visual anosognosia was accompanied by attention blindsight phenomena and signs of left spatial inattention (right-left strategy in tests of spatial exploration, left extinction to double side stimulation and increased reaction times for left stimuli).

These behavioral findings might support the hypothesis that two conditions might be necessary to deny blindness. The first is that some perceptual visual input, whenever wrong, partial or unconscious, must continue to be processed and the second is that some impairment in the associative areas of the right hemisphere (involved in spatial attention processing) precludes the identification of these visual remnants as abnormal or insufficient.

The occurrence of blindsight in the blind fields of hemianopic patients is a well documented phenomenon that encompasses a wide range of stimulus attributes (orientation, color, motion, etc) [11]. Blindisght is generally considered to be transmitted at the earliest stages of the vision process throughout sub-cortical retinotectal (i.e. superior colliculi) neuronal projections by the geniculate connections to the extra-striate cortical areas [12].

An unconscious extra-geniculo-striate visual perception might contribute, (for patients but also for normal subjects), to the belief that the visual stream is integer and continuous, whereas, actually, visual awareness probably results from the sum of different neural activated modules distributed in the brain in time and space [13].

Although attention blindsight could be a facilitating factor for anosognosia of a visual deficit, such phenomena also occur in patients who are aware of their visual field defects [14], a finding that points out the existence of supplementary mechanisms responsible for anosognosia of blindness. This assumption is further suggested by the evidence that patients with right parietal-occipital damage, left spatial neglect without visual field deficits are able to see objects on the left side of the space but remain unaware of this perception [15–19]. This last condition corresponds to that form of anosognosia that is specific to spatial neglect. Patients with spatial neglect are almost always unaware of their spatial bias [20,21].

If blindsight could be necessary to give continuity of vision to all patients with hemianopia (who do not have the feeling that the visual stream has been fragmented in half) anosognosia of a visual deficit may manifest only with supplementary damage to that neural network of spatial attention, which encroaches to the right hemisphere.

Table 1 summarizes the putative visual syndromes that occur as a consequence of trading off among unconscious perception (blindsight), spatial neglect, anosognosia and a visual field deficit.

Other authors previously suggested that spatial neglect and anosognosia might be not inextricable phenomena [22] and that unconscious perception and filling in phenomena may enhance confabulations, as well as false or abnormal perceptive motor processing or motor monitoring might trigger the false beliefs and confabulations on the paralyzed limb [23].

The association of anosognosia with spatial neglect and right hemispheric damage is strongly supported by cohort studies that demonstrated a significant higher frequency of anosognosia of hemianopia after right occipital or parieto-occipital stroke when spatial neglect was present [24].

Nevertheless, across different studies there are several reports of patients who presented anosognosia of hemianopia without spatial neglect [25–27]. However, in the above studies, patients with anosognosia of hemianopia without spatial neglect are the minority and the sensitivity of tests employed to detect spatial neglect might have been weak.

An alternative hypothesis is that anosognosia of patients with cortical blindness reflects the damage to pathways between the visual areas and areas mediating speech production or limbic areas engaged in memory processing, such that patients might be respectively unable to verbally report or to memorize what they see and produce confabulations.

The presence of pure alexia and severe memory disturbances in MP could be arguments for the visuo-verbal or limbic-visual disconnection hypothesis of anosognosia and confabulations.

However, as anosognosia of hemianopia has a strong association with right hemisphere lesions [28,25,26], the above hypothesis cannot exclude that the disconnected putative visual monitor area still belongs to the spatial attention network which is dominant in the right hemisphere.

The next step in verifying whether denial of a visual deficit manifests only in the presence of blindsight and signs related to left spatial neglect is to make a systematic survey of the signs of blindsight and spatial neglect in patients with occipital or occipital parietal lesions and contralateral hemianopia.
